# Supplementary material for: Cell-Based Influenza A/H1N1pdm09 Vaccine Viruses Containing Chimeric Hemagglutinin with Improved Membrane Fusion Ability
Source: Vaccines (Basel). 2020 Aug 19;8(3):458. doi: 10.3390/vaccines8030458 (PMC7565828; doi:10.3390/vaccines8030458)
Supplement: Supplementary file 1 [file vaccines-08-00458-s001.pdf]

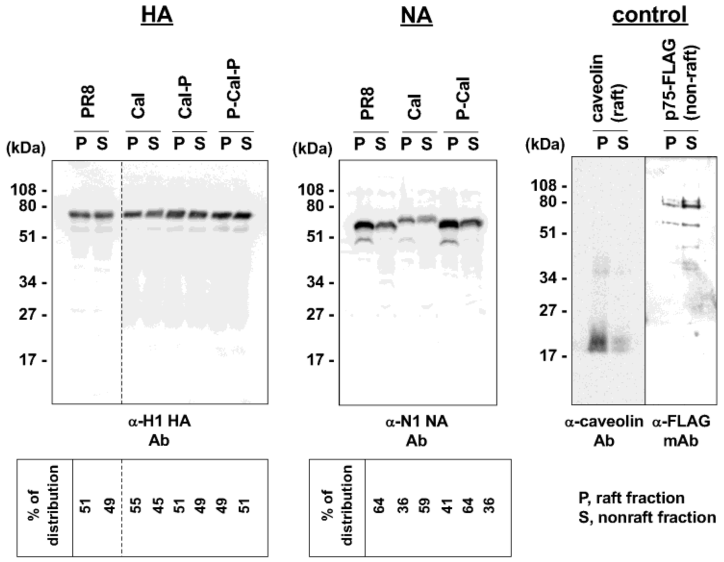

Figure S1. Affinity of HA and NA to lipid rafts. 293T cells were transfected with HA and NA expression plasmids. The cells were suspended with cold PBS and disrupted by sonication. The cell lysates were treated with 1% Triton X-100 at 4 °C for 30 min and centrifuged at 17,400×*g* at 4 °C for 30 min. The soluble (supernatant) and insoluble (precipitate) membrane fractions were subjected to western blotting with anti-H1 HA rabbit (left) and anti-NA sheep Ab (right). The band intensities were semi-quantified with an ImageJ software and are shown as % of distribution to the fractions. As controls, endogenous caveolin (a lipid raft-associated marker) was detected with anti-caveolin Ab, whereas neurotrophin receptor p75 (a non-raft marker) tagged with a FLAG sequence was exogenously expressed and detected anti-FLAG mAb.
